# Segmentation of dental cone‐beam CT scans affected by metal artifacts using a mixed‐scale dense convolutional neural network

**DOI:** 10.1002/mp.13793

**Published:** 2019-09-13

**Authors:** Jordi Minnema, Maureen van Eijnatten, Allard A. Hendriksen, Niels Liberton, Daniël M. Pelt, Kees Joost Batenburg, Tymour Forouzanfar, Jan Wolff

**Affiliations:** ^1^ Department of Oral and Maxillofacial Surgery/Pathology Amsterdam UMC and Academic Centre for Dentistry Amsterdam (ACTA) Vrije Universiteit Amsterdam Amsterdam Movement Sciences 3D Innovationlab 1081 HV Amsterdam The Netherlands; ^2^ Centrum Wiskunde & Informatica (CWI) 1090 GB Amsterdam The Netherlands; ^3^ Medical Technology Amsterdam UMC Vrije Universiteit Amsterdam 3D Innovationlab 1081 HV Amsterdam The Netherlands; ^4^ Department of Oral and Maxillofacial Surgery Division for Regenerative Orofacial Medicine University Hospital Hamburg‐Eppendorf 20246 Hamburg Germany; ^5^ Fraunhofer Research Institution for Additive Manufacturing Technologies IAPT Am Schleusengraben 13 21029 Hamburg Germany

**Keywords:** cone‐beam computed tomography (CBCT), image segmentation, metal artifacts

## Abstract

**Purpose:**

In order to attain anatomical models, surgical guides and implants for computer‐assisted surgery, accurate segmentation of bony structures in cone‐beam computed tomography (CBCT) scans is required. However, this image segmentation step is often impeded by metal artifacts. Therefore, this study aimed to develop a mixed‐scale dense convolutional neural network (MS‐D network) for bone segmentation in CBCT scans affected by metal artifacts.

**Method:**

Training data were acquired from 20 dental CBCT scans affected by metal artifacts. An experienced medical engineer segmented the bony structures in all CBCT scans using global thresholding and manually removed all remaining noise and metal artifacts. The resulting gold standard segmentations were used to train an MS‐D network comprising 100 convolutional layers using far fewer trainable parameters than alternative convolutional neural network (CNN) architectures. The bone segmentation performance of the MS‐D network was evaluated using a leave‐2‐out scheme and compared with a clinical snake evolution algorithm and two state‐of‐the‐art CNN architectures (U‐Net and ResNet). All segmented CBCT scans were subsequently converted into standard tessellation language (STL) models and geometrically compared with the gold standard.

**Results:**

CBCT scans segmented using the MS‐D network, U‐Net, ResNet and the snake evolution algorithm demonstrated mean Dice similarity coefficients of 0.87 ± 0.06, 0.87 ± 0.07, 0.86 ± 0.05, and 0.78 ± 0.07, respectively. The STL models acquired using the MS‐D network, U‐Net, ResNet and the snake evolution algorithm demonstrated mean absolute deviations of 0.44 mm ± 0.13 mm, 0.43 mm ± 0.16 mm, 0.40 mm ± 0.12 mm and 0.57 mm ± 0.22 mm, respectively. In contrast to the MS‐D network, the ResNet introduced wave‐like artifacts in the STL models, whereas the U‐Net incorrectly labeled background voxels as bone around the vertebrae in 4 of the 9 CBCT scans containing vertebrae.

**Conclusion:**

The MS‐D network was able to accurately segment bony structures in CBCT scans affected by metal artifacts.

## 1 INTRODUCTION

The spatial information embedded in medical three‐dimensional (3D) images is being increasingly used to personalize treatment by means of computer‐assisted surgery (CAS).^1^ This new field of medicine encompasses virtual surgical planning,^2^ 3D printing of personalized constructs,^3^ such as anatomical models, surgical saw guides, or implants,^4^ virtual and augmented reality^5^, ^6^ and robot‐guided surgery.^7^ The use of such emerging technologies in medicine has resulted in better treatment outcomes and a reduction in both operating times and costs.^3^, ^8^, ^9^ In recent years, CAS has reached a state of high technology readiness in maxillofacial surgery, where cone‐beam computed tomography (CBCT) is rapidly becoming the imaging modality of choice due to the low costs and radiation dose.^10^

**Figure 1:**
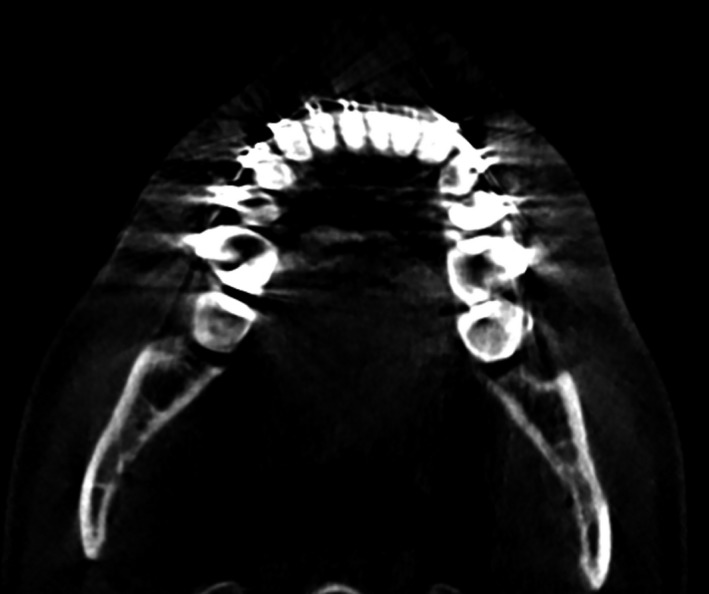
Example of metal artifacts in a cone‐beam computed tomography image of the mandible.

An essential step in the maxillofacial CAS workflow is the conversion of these CBCT images into a virtual 3D model of the anatomical region of interest.^11^ This conversion process requires accurate segmentation of bony structures in dental CBCT images.^12^ Image segmentation is, however, often impeded by metal artifacts.^13^ Such artifacts are caused by high‐density metal objects, such as amalgam fillings, crowns, dental implants, and retainers.^14^ The presence of high‐density metal objects in the radiation beam path induces photon starvation and scattering that lead to characteristic bright and dark streak artifacts in the resulting CBCT images (see Fig. 1).^15^ These streak artifacts can obscure anatomical structures and reduce the contrast between adjacent regions,^16^ and thereby impede the segmentation process of the teeth and bony structures in the mandible and maxilla.

To overcome these challenges, various metal artifact reduction (MAR) methods have been proposed. Such methods commonly aim to reduce metal artifacts during the reconstruction phase of CBCT scans.^17^ More specifically, an initial CBCT reconstruction is performed, followed by the segmentation of the metal structures and the removal of the segmented metal structures from the sinogram. Thereafter, a new reconstruction is performed based on the corrected sinogram, which results in a reduced incidence of metal artifacts in the reconstructed CBCT scan.^18^ However, the performance of such MAR methods depends strongly on the quality of the initial metal artifact segmentation,^19^ and is often limited by the introduction of secondary artifacts^18^, ^20^ and incomplete metal artifact correction.^21^ As a consequence, metal artifacts remain a challenge in CAS.

In recent years, deep learning has been increasingly used for MAR. The majority of these approaches are based on convolutional neural networks (CNNs). CNNs can learn to extract information from a large number of training images to perform certain tasks in the MAR workflow, such as CBCT sinogram correction.^22^, ^23^, ^24^ In a recent study by Zhang and Yu (2018), a CNN‐based MAR framework was developed that fused the information from original and corrected MDCT images to suppress metal artifacts.^25^ These corrected MDCT images were obtained by combining multiple conventional MAR methods. A major drawback of such MAR frameworks is that they first need to be trained using two sets of images of the same patient — one set of artifact free images and one set of images affected by artifacts. Since such paired datasets are often unavailable in clinical settings, most deep learning‐based MAR methods rely on mathematical simulations of metal artifacts that typically do not fully represent the photon and detector physics of individual MDCT or CBCT scanners.

Instead of relying solely on such mathematical simulations, it is also possible to use deep learning for MAR during the CBCT image segmentation step. A major advantage of CNNs is that they can be efficiently trained using high quality “gold standard” CBCT segmentations of teeth and bony structures created by human experts during CAS. To date, a variety of CNN architectures have been proposed for medical image segmentation.^26^ However, since it is relatively difficult to acquire a sufficient number of gold standard segmentations in clinical settings, it is important to choose a CNN architecture with few trainable parameters that can be trained using few datasets. Therefore, in this study, the authors for the first time employed a novel mixed‐scale dense CNN (MS‐D network) architecture^27^ to segment dental CBCT scans affected by metal artifacts. Furthermore, the performance of this MS‐D network was compared with two state‐of‐the‐art CNN architectures, namely U‐Net^28^ and ResNet.^29^ In addition, a clinical snake evolution algorithm^30^ that is commonly used for medical image segmentation was evaluated.

Specifically, the main contributions of this study are as follows:

1. CNNs were used to deal with metal artifacts in dental CBCT scans during image segmentation, rather than image reconstruction.
2. A novel mixed‐scale dense CNN was trained on a relatively small dataset of dental CBCT images.
3. The mixed‐scale dense CNN resulted in comparable segmentation performances as U‐Net and ResNet CNN architectures, while using far fewer trainable parameters.
4. All CNNs outperformed a widely‐used clinical snake evolution method.

## 2 Materials and Methods

### 2.A. Data acquisition

A total of 20 dental CBCT scans that had been heavily affected by metal artifacts caused by dental restorations and appliances were used in this study. Of these CBCT scans, 2 were used for validation (see section “Code implementation”) and 18 were used for training (see section “Evaluation”). All scans were obtained on a Vatech PaX‐Zenith3D (Vatech, Gyeonggi‐do, South‐Korea) CBCT scanner using a tube voltage of 105 kVp, a tube current of 6 mA and an isotropic voxel size of 0.2 mm. Each CBCT scan was cropped to a confined region of interest that included the lower part of the maxilla, the mandible and both condyles, resulting in variable scan dimensions ranging from 800 × 412 × 190 (patient 9) to 1000 × 724 × 383 (patient 10). All CBCT scans were normalized by subtracting the mean voxel value of the training CBCT scans and dividing the resulting values by the standard deviation.

In order to train a CNN for bone segmentation in these CBCT scans, gold standard segmentation labels were required. These gold standard labels were created by segmenting all CBCT scans using global thresholding, followed by extensive manual postprocessing by an experienced medical engineer using Mimics software (Mimics v20.0, Materialise, Leuven, Belgium). This postprocessing step was necessary to remove the noise and metal artifacts caused by dental fillings and appliances. This task took approximately 2 h per scan to complete.

### 2.B. CNN architecture

In this study, we used a mixed‐scale dense CNN (MS‐D network) architecture originally proposed by Pelt and Sethian.^27^ This network architecture combines small‐ and large‐scale features with far fewer trainable parameters compared with state‐of‐the‐art U‐Net architectures.^28^ These properties enable an MS‐D network to be trained more efficiently and reduce the risk of overfitting.^27^

**Figure 2:**
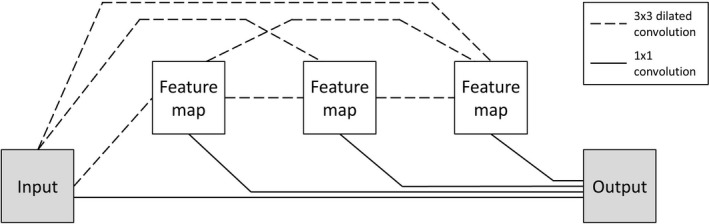
Schematic representation of a mixed‐scale dense network architecture with three convolutional layers.

A schematic overview of an MS‐D network architecture with three convolutional layers is presented in Figure 2. Note that the MS‐D network architecture used in this study comprised 100 convolutional layers. Each convolutional layer performs a convolutional operation on its input to produce an intermediate image, also known as a *feature map*. All feature maps are used to compute the final output segmentation.

A feature map $z_{i}$ in convolutional layer i is calculated as

(1) $$z_{i}=\sigma \left(g_{i}\left(\left\{z_{0},\ldots ,z_{i-1}\right\}\right)+b_{i}\right)$$

where σ is a rectified linear unit (ReLU) activation function^31^ and $b_{i}$ is a constant bias term. The function *g_i_* performs a 2D 3 × 3 dilated convolution $D_{h,s}$ on all previously computed feature maps $\left\{z_{0},\cdots ,z_{i-1}\right\}$ and sums the resulting feature maps in a pixel‐wise manner, giving

(2) $$g_{i}\left(\left\{z_{0},\ldots ,z_{i-1}\right\}\right)=\sum_{j=0}^{i-1}D_{h_{\mathrm{ij}},s_{i}}z_{j},$$

where *h* is a convolutional kernel and *s* is the dilation factor. In a dilated convolution, a kernel *h* is expanded by a dilation factor *s* and filled with zeros at distances that are not a multiple of *s* voxels from the kernel center. Thus, by increasing the dilation factor, the MS‐D network is able to detect large features^27^ without increasing the number of kernel weights.^32^ In this study, the dilation factor was initialized as 1 in the first convolutional layer, and then increased by 1 in each subsequent convolutional layer. After 10 convolutional layers, the dilation factor was reset to 1 and the process was repeated. This enabled the MS‐D network to extract mixed‐scale features from the input CBCT slices. In addition, all dilated convolutions were performed using reflective boundaries. As a result, the size and shape of all feature maps remained equal to those of the initial input. The major advantage of equally sized feature maps is that the convolutional layers are not restricted to using only the feature map of the previous layer to compute a new feature map. Instead, all previously computed feature maps, including the initial input, are used to compute a new feature map, resulting in a densely connected network (Fig. 2).

The output *y* of an MS‐D network is computed by applying 1 × 1 convolutional kernels $w_{i}$ to all previously computed feature maps $\left\{z_{0},\cdots ,z_{i-1}\right\}$, adding a constant bias term *b* and applying a softmax activation function $\sigma^{{}^{\prime }}$. This can be written as follows:

(3) $$y=\sigma^{\prime }\left(\sum_{i}w_{i}z_{i}+b\right).$$

Since *y* is a continuous variable with values between 0 and 1, a cut‐off value was required to obtain a binary segmentation (i.e., “bone” or “background”). In the present study, we treated this cut‐off value as an additional hyper‐parameter of the MS‐D network.

### 2.C. Implementation and training details

The hyper‐parameters of the MS‐D network, that is, the number of layers and the cut‐off value, were determined by validating the network on two CBCT scans. During these validation experiments, the number of layers was varied between 30 and 150 (30, 50, 80, 100 and 150), and the cut‐off value was varied between 0.1 and 0.9 with a step size of 0.2. Optimal performance of the MS‐D network was achieved using 100 convolutional layers and a cut‐off value of 0.7. The validation dataset was also used to find the optimal number of epochs (10) for training.

The MS‐D network was implemented in Python (version 3.6.1) using Pytorch (version 0.3.1). Training of the MS‐D network was performed on 2D axial CBCT slices using a batch size of 1 and the default Adam optimizer^33^ on a Linux desktop computer (HP Workstation Z840) with 64 GB RAM, a Xeon E5‐2687 v4 3.0 GHZ CPU and a GTX 1080 Ti GPU card. Training took approximately 5 h for each epoch.

### 2.D. Evaluation

The segmentation performance of the MS‐D network was evaluated using the 18 CBCT scans available for training (see Section 2.A). A leave‐2‐out scheme^34^ was used so that 16 of the 18 training CBCT scans were alternately used for training and 2 for testing. As a clinical benchmark, these 18 CBCT scans were also segmented using a snake evolution algorithm that is commonly used for various clinical segmentation purposes.^35^, ^36^, ^37^, ^38^ This algorithm is available in the open‐source ITK‐SNAP software package^30^ and requires an initial segmentation using global thresholding, followed by selection of seed points in the region of interest (i.e., bone).

In addition, the performance of the MS‐D network was compared to two state‐of‐the‐art CNN architectures available on Github, namely U‐Net^39^ and ResNet.^40^ The U‐Net used in this study is comparable to the one described by Ronneberger et al.,^28^ except that our implementation performed batch normalization^41^ after each ReLU and used reflection padding on images of which the dimensions were not divisible by 16. The ResNet used in this study was a residual network comprising 50 layers as described by He et al.^29^ Both CNNs were trained using 4 epochs and a cut‐off value of 0.3.

The segmentation performance of all three CNNs and the clinical snake evolution algorithm was evaluated using the Dice similarity coefficient (DSC). The DSC indicates the overlap between a segmented CBCT scan and the corresponding gold standard segmentation. This can be written as follows:

(4) $$DSC=\frac{2TP}{2TP+FP+FN},$$

where TP is the number of true positives, FP is the number of false positives and FN is the number of false negatives.

All segmented CBCT scans and corresponding gold standard segmentations were subsequently converted into virtual 3D models in the standard tessellation language (STL) file format using 3D Slicer software.^42^, ^43^ The resulting STL models were geometrically compared with the corresponding gold standard STL models using the surface comparison function in GOM Inspect**®** software (GOM Inspect 2018, GOM GmbH, Braunschweig, Germany). Signed deviations between −5.0 and +5.0 mm were measured between the acquired STL models and the gold standard STL models. The mean absolute deviations (MADs) were calculated for all STL models.

## 3 RESULTS

**Figure 3:**
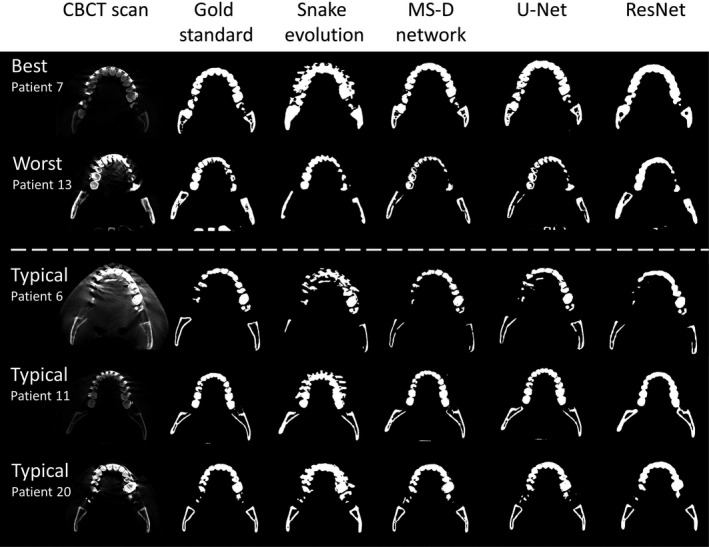
Examples of the best (patient 7) and worst (patient 13) mixed‐scale dense (MS‐D) network performances and three typical examples (patients 6, 11, and 20). Each example comprises an axial cone‐beam computed tomography slice, the gold standard (manual) segmentation, and the segmentations acquired using the snake evolution algorithm, MS‐D network, U‐Net and ResNet.

In all CBCT scans affected by metal artifacts, the MS‐D network resulted in fewer erroneously labeled voxels in the dental region than the snake evolution algorithm (Fig. 3). Moreover, the MS‐D network resulted in a more complete segmentation of the condyles and the rami than the snake evolution algorithm in 13 of the 18 CBCT scans (Fig. 3). Furthermore, in 8 out of 9 CBCT scans that contained parts of the vertebrae, the MS‐D network segmented these vertebrae, whereas the snake evolution algorithm incorrectly labeled the vertebrae as the background in all 9 CBCT scans. Quantitatively, the snake evolution algorithm and the MS‐D network resulted in a mean DSC of 0.78 ± 0.07 and 0.87 ± 0.06, respectively (Table 1). U‐Net and ResNet resulted in mean DSCs of 0.87 ± 0.07 and 0.86 ± 0.05, respectively.

**Table 1:** Dice similarity coefficients (DSCs) of all cone‐beam computed tomography scans segmented using the snake evolution algorithm, MS‐D network, U‐Net and ResNet.

Patient ID	Snake evolution	MS‐D network	U‐Net	ResNet
1	Validation set	Validation set	Validation set	Validation set
2	Validation set	Validation set	Validation set	Validation set
3	0.67	0.89	0.86	0.82
4	0.72	0.85	0.79	0.75
5	0.60	0.75	0.78	0.78
6	0.80	0.85	0.88	0.82
7	0.76	0.94	0.93	0,90
8	0.83	0.92	0.91	0.90
9	0.80	0.89	0.87	0.78
10	0.90	0.92	0.92	0.90
11	0.84	0.92	0.92	0.85
12	0.84	0.78	0.69	0.90
13	0.73	0.73	0.78	0.82
14	0.86	0.83	0.88	0.90
15	0.75	0.91	0.90	0.88
16	0.77	0.91	0.92	0.90
17	0.76	0.86	0.91	0.86
18	0.81	0.88	0.94	0.92
19	0.86	0.90	0.87	0.82
20	0.79	0.91	0.90	0.90
Mean	0.78 ± 0.07	0.87 ± 0.06	0.87 ± 0.07	0.86 ± 0.05

**Figure 4:**
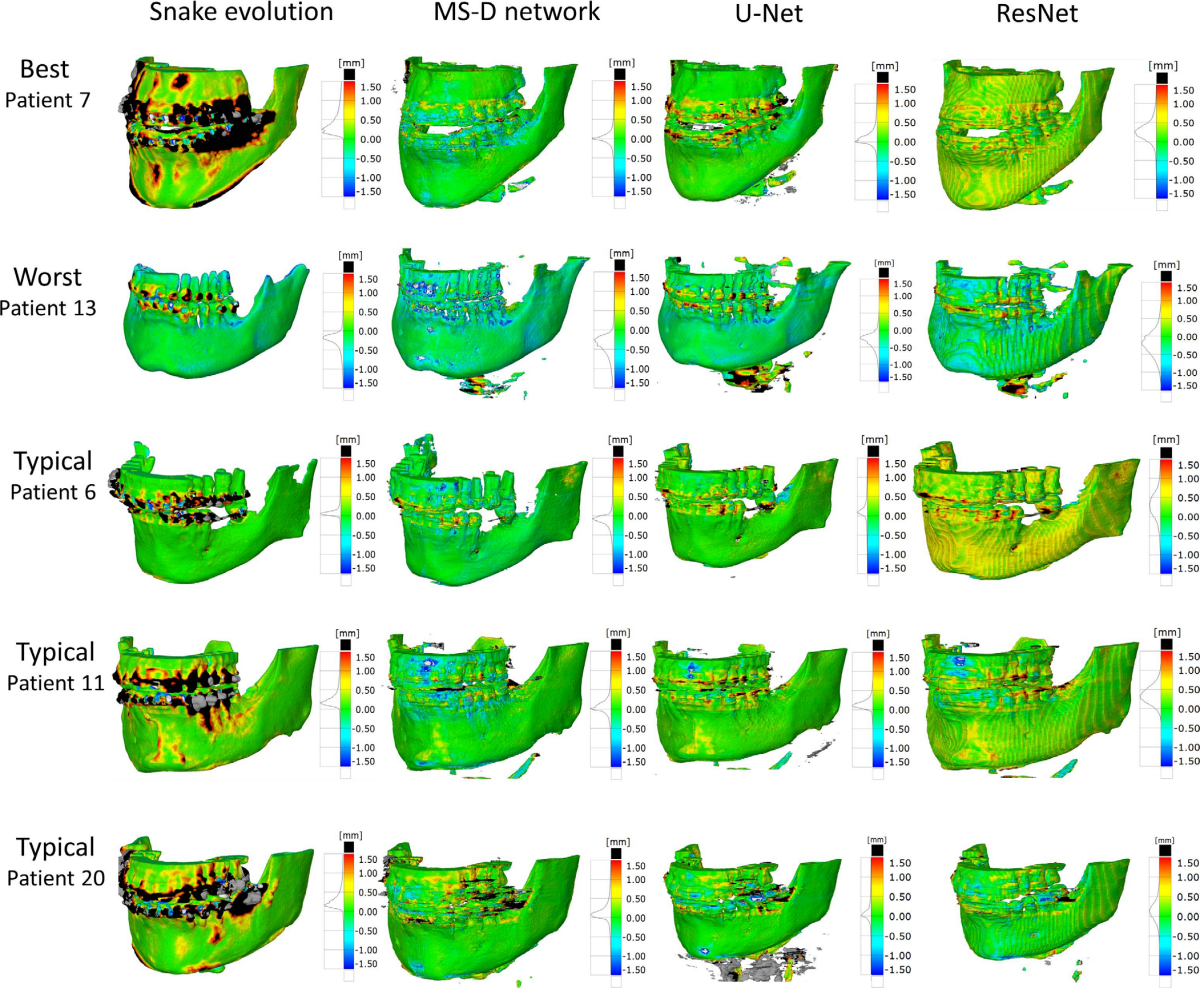
Color maps of the surface deviations of five standard tessellation language (STL) models acquired using the snake evolution algorithm, mixed‐scale dense network, U‐Net and ResNet. All depicted surface deviations were calculated with respect to the corresponding gold standard STL model. [Color figure can be viewed at http://wileyonlinelibrary.com]

Generally, all STL models acquired using the CNNs, i.e., the MS‐D network, U‐Net and ResNet, contained fewer outliers in the dental region than the STL models acquired using the snake evolution algorithm (Fig. 4). However, in contrast to the MS‐D network, the ResNet introduced wave‐like artifacts in all 18 STL models (Fig. 4), whereas the U‐Net incorrectly labeled background voxels as bone around the vertebrae in 4 of the 9 CBCT scans containing vertebrae (Fig. 4; patients 7,13 and 20).

**Figure 5:**
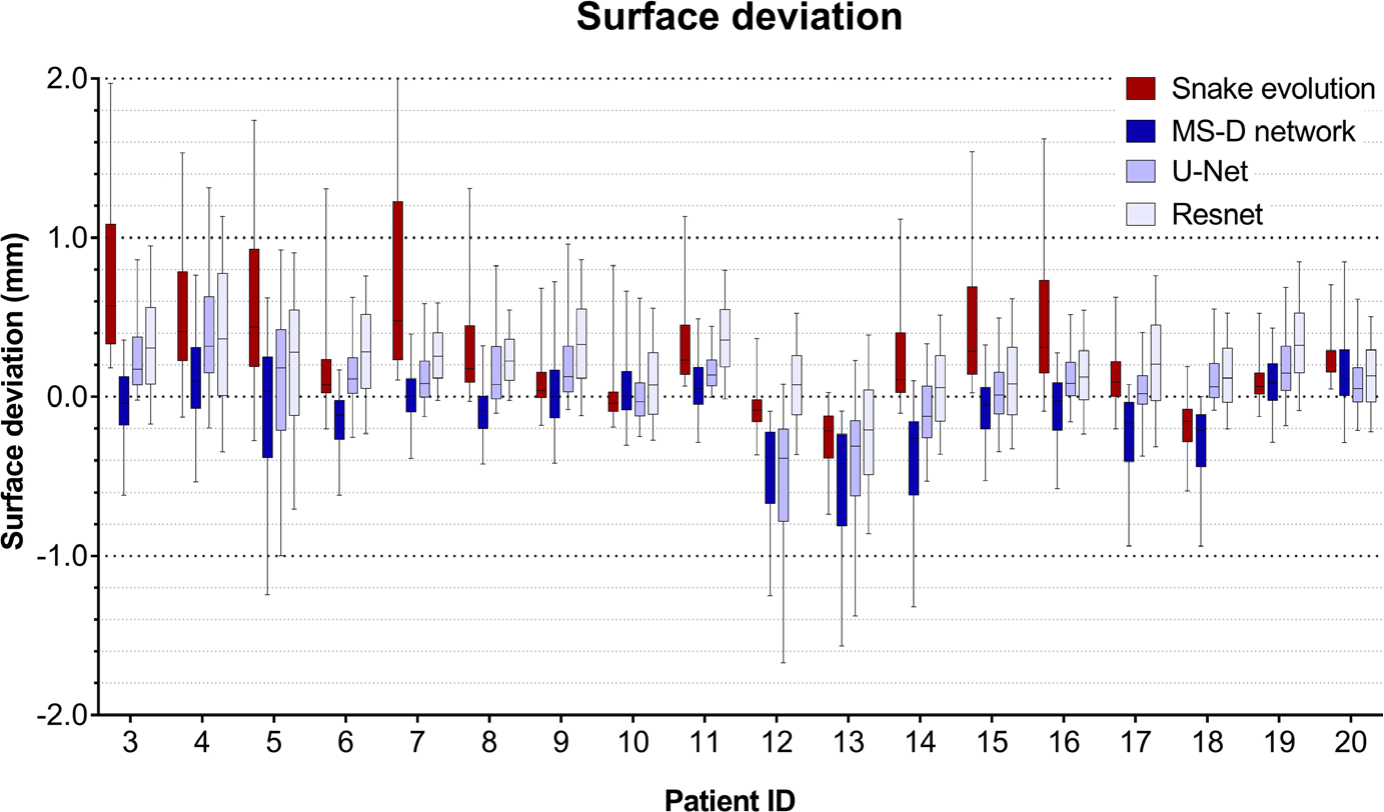
Box and whisker plot of the surface deviations between the standard tessellation language (STL) models acquired using the snake evolution algorithm, MS‐D network, U‐Net, ResNet, and the corresponding gold standard STL models. The boxes represent the interquartile range and the whiskers represent the 10th and 90th percentiles of the surface deviations. [Color figure can be viewed at http://wileyonlinelibrary.com]

Figure 5 visualizes the surface deviations between all STL models and their corresponding gold standard STL models. In 11 of the 18 patients, the 10–90 percentile range acquired using the snake evolution algorithm was larger than those acquired using the CNNs. When compared with the gold standard STL models, the STL models acquired using the MS‐D network resulted in a mean MAD of 0.44 ± 0.13 mm; whereas the STL models acquired using the snake evolution algorithm resulted in a mean MAD of 0.57 ± 0.22 mm. The STL models acquired using U‐Net and ResNet resulted in mean MADs of 0.43 ± 0.16 mm and 0.40 ± 0.12 mm, respectively.

## 4 DISCUSSION

High‐density metal fillings and appliances are very common in the oral cavity. For example, more than half of the American population has at least one dental filling and approximately 25% are estimated to have more than 7 fillings.^44^ Consequently, metal artifacts caused by such objects remain a challenge in CBCT imaging. Such artifacts can obscure bony regions in the mandible and maxilla and can lead to inaccuracies and time constraints during the image segmentation process required for computer‐assisted maxillofacial surgery.

All CNNs trained in this study (MS‐D network, U‐Net and ResNet) were able to segment bony structures in CBCT scans and classify metal artifacts as background more accurately than the current clinical benchmark, i.e., the snake evolution algorithm (Fig. 3 and Table 1). This finding is likely due to the CNNs' ability to learn characteristic features that distinguish bone from metal artifacts. The snake evolution algorithm, on the other hand, is a model‐driven segmentation method that is solely based on identifying intensity gradients in images. Although such intensity‐based image segmentation methods generally perform well in identifying the edges of bony structures in CBCT images,^35^ they tend to fail in the presence of metal artifacts due to the introduction of strong intensity gradients in the reconstructed CBCT images.

The DSCs found in our study (Table 1) are comparable to those reported by Wang et al. (2015), who used a prior‐guided random forest to segment the maxilla and mandible in 30 CBCT scans and reported a mean DSC of 0.91 ± 0.03 for the maxilla and 0.94 ± 0.02 for the mandible.^45^ However, their dataset only included 4 CBCT scans that were affected by metal artifacts. Evain et al. (2017) recently developed a graph‐cut approach for the segmentation of individual teeth.^46^ Although their algorithm achieved a high mean DSC of 0.958 ± 0.023, they also reported that false edges were induced in images affected by metal artifacts.^46^

As an additional evaluation step in our study, all segmented CBCT scans were converted into STL models and geometrically compared with the corresponding gold standard STL models. Interestingly, fewer outliers were observed in the STL models acquired using the CNNs than in the STL models acquired using the snake evolution algorithm (Figs. 4 and 5). The MADs acquired in the present study are smaller than those obtained by Lamecker et al. (2006), who developed a statistical shape model for the segmentation of the mandible in CBCT scans and found mean surface deviations larger than 1 mm, even though they excluded all teeth from statistical analysis due to severe metal artifacts.^47^ The MADs obtained in this study are, however, higher than those reported by Gan et al. (2014), who segmented individual teeth in CBCT scans using a level‐set method and achieved a MAD of 0.3 ± 0.08 mm.^48^ Nevertheless, it must be noted that Gan et al. did not include any CBCT scans affected by metal artifacts because their level‐set algorithm failed to identify teeth contours in these scans.

The novel MS‐D network resulted in accurate segmentations that were comparable to those achieved by U‐Net and ResNet, using fewer trainable parameters (Table 2). Reducing the number of parameters is crucial in clinical settings since it minimizes the risk of overfitting^27^ and prevents common deep learning issues such as vanishing gradients and local minima.^49^ Another major advantage of MS‐D networks over U‐Net and ResNet is the use of dilated convolutional kernels instead of standard convolutional kernels. This allows MS‐D networks to learn which combinations of dilations are most suited to solve the task at hand and offers the unique possibility to use the same MS‐D network architecture for a broad range of different applications such as segmenting organelles in microscopic cell images,^27^ image denoising^27^ and improving the resolution of tomographic reconstructions.^50^ Finally, all layers of an MS‐D network are interconnected using the same set of standard operations [see Section 2, Eqs. (1) and (2)], which greatly simplifies implementation and training of an MS‐D network in clinical settings.^27^

**Table 2:** The number of trainable parameters used by the MSD network, U‐Net and ResNet.

CNN model	Number of trainable parameters
MS‐D network	45,756
U‐Net	14,787,842
ResNet	32,940,996

Another interesting finding in this study was that the MS‐D network was able to accurately segment bony regions that were not affected by metal artifacts, such as the medial parts of the rami, the condyles and the vertebrae (Fig. 3; patients 6 and 13). On the other hand, the segmentations obtained using ResNet demonstrated less anatomical details (Figs. 3 and 4), which can result in ill‐fitting personalized constructs during CAS.^4^ Furthermore, the segmentations obtained using U‐Net were less accurate in the vicinity of the vertebrae when compared to those obtained using the MS‐D network. A possible explanation for this phenomenon is that the MS‐D network was better capable to learn features of relatively rare structures in the training dataset such as the vertebrae. These results demonstrate that the MS‐D network is well suited for “real‐world” clinical segmentation purposes.

An important advantage of all three CNNs evaluated in this study over alternative clinical segmentation methods is the short computational time required for image segmentation. More specifically, all three CNNs automatically segmented each CBCT scan in <5 min. In comparison, the semi‐automatic clinical snake evolution algorithm segmented a single CBCT scan in 20 min to 1 h. All CNN segmentation times found in this study are markedly quicker than the atlas‐based method described by Wang et al. (2015) that segmented a single CBCT scan in 5 h.^45^ Moreover, a previously published patch‐based CNN for MDCT image segmentation of the skull took approximately 1 h to segment a single MDCT scan.^51^ The short segmentation times of the CNNs in this study were primarily due to their fully‐convolutional nature that allows the CNNs to segment CBCT images using far fewer convolutional operations than patch‐based CNNs.^26^

Taking the aforementioned advantages in terms of performance and speed into account, deep learning is now coming of age for medical image segmentation, especially with advanced architectures such as the MS‐D network. The next step toward making deep learning‐based solutions available for challenging image segmentation tasks in CAS would be to develop, test and certify interactive plug‐ins for medical image processing software packages.

### 4.1 Limitations

One challenge that all supervised deep learning algorithms have in common is the overall accuracy of the gold standard segmentations. Especially the presence of metal artifacts can negatively influence the judgements of experienced medical engineers and subsequently affect the quality of their gold standard segmentations. Furthermore, the process of creating sufficient gold standard segmentations can be very time‐consuming. One solution could be to adopt an iterative training strategy in which a pretrained CNN is used to perform an initial segmentation of a CBCT scan, after which a medical engineer only has to correct the errors and retrain the CNN. Another interesting direction for future research is the potential use of 3D CNNs due to the 3D characteristics of metal artifacts in dental CBCT scans.

## 5 CONCLUSION

This study presents a mixed‐scale dense CNN (MS‐D network) to segment teeth and bony structures in CBCT images heavily affected by metal artifacts. Experimental results demonstrated that the segmentation performance of the MS‐D network was comparable to those of state‐of‐the‐art U‐Net and ResNet CNN architectures, while preserving more anatomical details in the resulting STL models and using fewer trainable parameters. Moreover, all CNNs outperformed a commonly used clinical snake evolution algorithm. These promising results show that deep learning offers unique possibilities to eliminate the inaccuracies caused by metal artifacts in the CAS workflow.

## ETHICAL CONSIDERATIONS

This study followed the principles of the Helsinki Declaration and was performed in accordance with the guidelines of the Medical Ethics Committee of the Amsterdam UMC. The Dutch Medical Research Involving Human Subjects Act (WMO) did not apply to this study (Ref: 2017.145).
